# A systematic review and meta-analysis of obstetric and maternal outcomes after prior uterine artery embolization

**DOI:** 10.1038/s41598-021-96273-z

**Published:** 2021-08-19

**Authors:** Shinya Matsuzaki, Misooja Lee, Yoshikazu Nagase, Mariko Jitsumori, Satoko Matsuzaki, Michihide Maeda, Tsuyoshi Takiuchi, Aiko Kakigano, Kazuya Mimura, Yutaka Ueda, Takuji Tomimatsu, Masayuki Endo, Tadashi Kimura

**Affiliations:** 1 Department of Obstetrics and Gynecology, Osaka University Graduate School of Medicine, 2-2 Yamadaoka, Suita, Osaka 565-0871 Japan; 2 Department of Gynecology, Osaka International Cancer Institute, Osaka, Japan; 3 Division of Gynecologic Oncology, Department of Obstetrics and Gynecology, University of Southern California, Los Angeles, CA USA; 4 Department of Obstetrics and Gynecology, Osaka General Medical Center, Osaka, Japan; 5 Department of Obstetrics and Gynecology, National Cerebral and Cardiovascular Center, Osaka, Japan; 6 Department of Health Science, Osaka University Graduate School of Medicine, Osaka, Japan

**Keywords:** Outcomes research, Risk factors

## Abstract

This study aimed to review the obstetric complications during subsequent pregnancies after uterine artery embolization (UAE) for postpartum hemorrhage (PPH) by exploring the relationship between prior UAE and obstetric complications through a meta-analysis. We conducted a systematic literature review through March 31, 2021, using PubMed, Scopus, and the Cochrane Central Register of Controlled Trials in compliance with the PRISMA guidelines and determined the effect of prior UAE for PPH on the rate of placenta accreta spectrum (PAS), PPH, placenta previa, hysterectomy, fetal growth restriction (FGR), and preterm birth (PTB). Twenty-three retrospective studies (2003–2021) met the inclusion criteria. They included 483 pregnancies with prior UAE and 320,703 pregnancies without prior UAE. The cumulative results of all women with prior UAE indicated that the rates of obstetric complications PAS, hysterectomy, and PPH were 16.3% (34/208), 6.5% (28/432), and 24.0% (115/480), respectively. According to the patient background-matched analysis based on the presence of prior PPH, women with prior UAE were associated with higher rates of PAS (odds ratio [OR] 20.82; 95% confidence interval [CI] 3.27–132.41) and PPH (OR 5.32, 95% CI 1.40–20.16) but not with higher rates of hysterectomy (OR 8.93, 95% CI 0.43–187.06), placenta previa (OR 2.31, 95% CI 0.35–15.22), FGR (OR 7.22, 95% CI 0.28–188.69), or PTB (OR 3.00, 95% CI 0.74–12.14), compared with those who did not undergo prior UAE. Prior UAE for PPH may be a significant risk factor for PAS and PPH during subsequent pregnancies. Therefore, at the time of delivery, clinicians should be more attentive to PAS and PPH when women have undergone prior UAE. Since the number of women included in the patient background-matched study was limited, further investigations are warranted to confirm the results of this study.

## Introduction

Postpartum hemorrhage (PPH) occurs in approximately 5% of deliveries, and severe PPH has led to approximately 140,000 annual maternal deaths worldwide^1–5^. According to the American College of Obstetricians and Gynecologists, PPH results in a cumulative blood loss of ≥ 1000 mL or is characterized by the presence of symptoms of hypovolemia related to blood loss within 24 h after vaginal or cesarean delivery^6^. First-line treatment for PPH includes pharmacological measures, intrauterine tamponade, uterine artery ligation, and uterine compression sutures; uterine artery embolization (UAE) is performed for women with treatment-refractory severe PPH^6–12^. If these procedures cannot achieve homeostasis, then hysterectomy is performed.

UAE is a useful alternative to hysterectomy for managing severe PPH^6^. It is an effective and minimally invasive procedure with feasible side effects and a consistent success rate of more than 90% for achieving hemostasis. Therefore, UAE is an essential procedure for treating severe PPH. Moreover, UAE for PPH has feasible short-term and long-term adverse effects^13–16^. According to a systematic review, the fertility rate after UAE for patients attempting another pregnancy is 70–80%^17^.

Prior UAE appears to be associated with an increased rate of various obstetric complications, such as placenta accreta spectrum (PAS), placenta previa, and PPH, during subsequent pregnancies^18–21^. Nevertheless, the rates of fetal growth restriction (FGR) and preterm birth (PTB) have not been sufficiently studied. Knowing the risks of maternal outcomes and obstetric outcomes of subsequent pregnancies after UAE may be helpful for its antenatal diagnosis and treatment involving multidisciplinary care^22,23^. Notably, recent systematic reviews have reported that the antenatal diagnosis of PAS is associated with improved maternal outcomes^24,25^. This study aimed to determine the effect of prior UAE on obstetric complications, including PAS, and maternal outcomes of subsequent pregnancies.

## Materials and methods

### Systematic literature review approach

A systematic review was performed to review the effect of prior UAE on subsequent pregnancies. The outcomes of interest were the rates of PAS, hysterectomy, PPH, placenta previa, FGR, PTB, and UAE, and the maternal outcomes (rates of urinary tract injury, infection, and transfusion). In compliance with the Preferred Reporting Items for Systematic Reviews and Meta-Analyses (PRISMA) guidelines, 2020 edition^26^, a systematic search was performed using PubMed (sorting by most recent), Scopus, and the Cochrane Central Register of Controlled Trials (CENTRAL) from inception to March 31, 2021, using MeSH terms (if applicable) and text words for the concepts “Uterine Artery Embolization” and “pregnancy” (see Supplemental Table S1 for complete search strategies). There were no date, language, or other restrictions.

### Eligibility criteria, information sources, and search strategy

The concepts “Uterine Artery Embolization” and “pregnancy” were searched using the text words listed in Supplemental Table S1. These key words were entered in PubMed, Scopus, and CENTRAL to identify studies that examined the association between prior UAE and the outcomes of interest (MeSH terms were used for the PubMed and CENTRAL searches).

### Study selection

The inclusion criteria, which were based on the Patient/Population, Intervention, Comparator, Outcome, Study (PICOS) process are shown in Supplemental Table S2. Studies were selected according to the following inclusion criteria: (1) the effect of prior UAE on the risk of the outcome of interest was examined; (2) PPH was controlled by UAE during prior pregnancy; (3) a comparative study of the outcome of interest was also included (UAE versus non-UAE); and (4) at least four subsequent pregnancies were included.

The exclusion criteria were as follows: (1) insufficient information about the outcomes of interest; (2) included > 10% of women who underwent UAE for uterine myoma; (3) included > 10% of women who underwent UAE for early pregnancy; (4) articles were not written in English; and (5) conference abstracts, case reports, case series, and reviews.

Studies were identified by screening the titles, abstracts, and full texts of the relevant articles. All titles, abstracts, and full texts were independently screened by the authors (Sh.M. and L.M.).

### Data extraction

Two authors (Sh.M. and L.N.) independently extracted the data and recorded the following variables: UAE type; year of study; first author’s name; study location; number of included cases; PAS and PPH definitions; obstetric outcomes (rates of PAS, hysterectomy, PPH, placenta previa, FGR, and PTB); and maternal outcomes (rates of urinary tract injury, infection, and transfusion). Information regarding embolic agents for UAE was also collected. Since the preparation of agents may affect the quantity of embolic agents, information regarding the agent preparation was also collected^27^.

### Outcome measure analysis and assessment of the risk of bias

Our primary objective was to assess the effect of prior UAE on the rates of PAS and hysterectomy during subsequent pregnancies. One secondary objective was divided into two sub-objectives, namely examining the effect of prior UAE on the rate of PPH and examining the effect of prior UAE on other obstetric complications such as placenta previa, FGR, and PTB. Another secondary objective was the assessment of the effect of prior UAE on maternal outcomes such as the rates of urinary tract injury, infection, and transfusion. The risk of bias was assessed using the Risk Of Bias in Nonrandomized Studies of Interventions (ROBINS-I) tool^28–30^.

### Meta-analysis plan

Using the eligible study data, the risks of the outcomes of interest (PAS, hysterectomy, PPH, placenta previa, FGR, PTB, and UAE during subsequent pregnancies after UAE) were computed using the 95% confidence intervals (CIs) of the reported values to estimate the odds ratios (ORs) for the rate of these outcomes. The heterogeneity of the studies was examined using *I*^2^ statistics to measure the percentage of total variation across these studies. According to the *Cochrane Handbook for Systematic Reviews of Interventions* (version 6.0), heterogeneity was assessed based on the *I*^*2*^ value with the following modifications: 0% to < 30%, low heterogeneity; 30–60%, moderate heterogeneity; 50–90%, substantial heterogeneity; and 75–100%, considerable heterogeneity^31^.

We conducted the meta-analysis and created all graphics using RevMan ver. 5.4.1 software (Cochrane Collaboration, Copenhagen, Denmark). For consistency, data regarding all outcomes (continuous and bivariate) were entered into the software so that negative effect sizes or relative risks < 1 favored active intervention. During the pooled analysis, a fixed-effect analysis was performed if the heterogeneity of the studies was considered low; a random-effect analysis was performed if the heterogeneity of the studies was considered moderate to considerable.

### Statistical analysis

Differences in baseline demographics between the two groups were assessed with the Fisher exact test or chi-square as appropriate^32^. All statistical analyses were based on two-sided hypotheses, and *P* < 0.05 was considered statistically significant. Statistical Package for Social Sciences (IBM SPSS, version 27.0, Armonk, NY, USA) was used for the analysis.

### Ethical approval

The approval of Institutional Review Board exempted the use of publicly available data.

## Results

### Study selection

**Figure 1:**
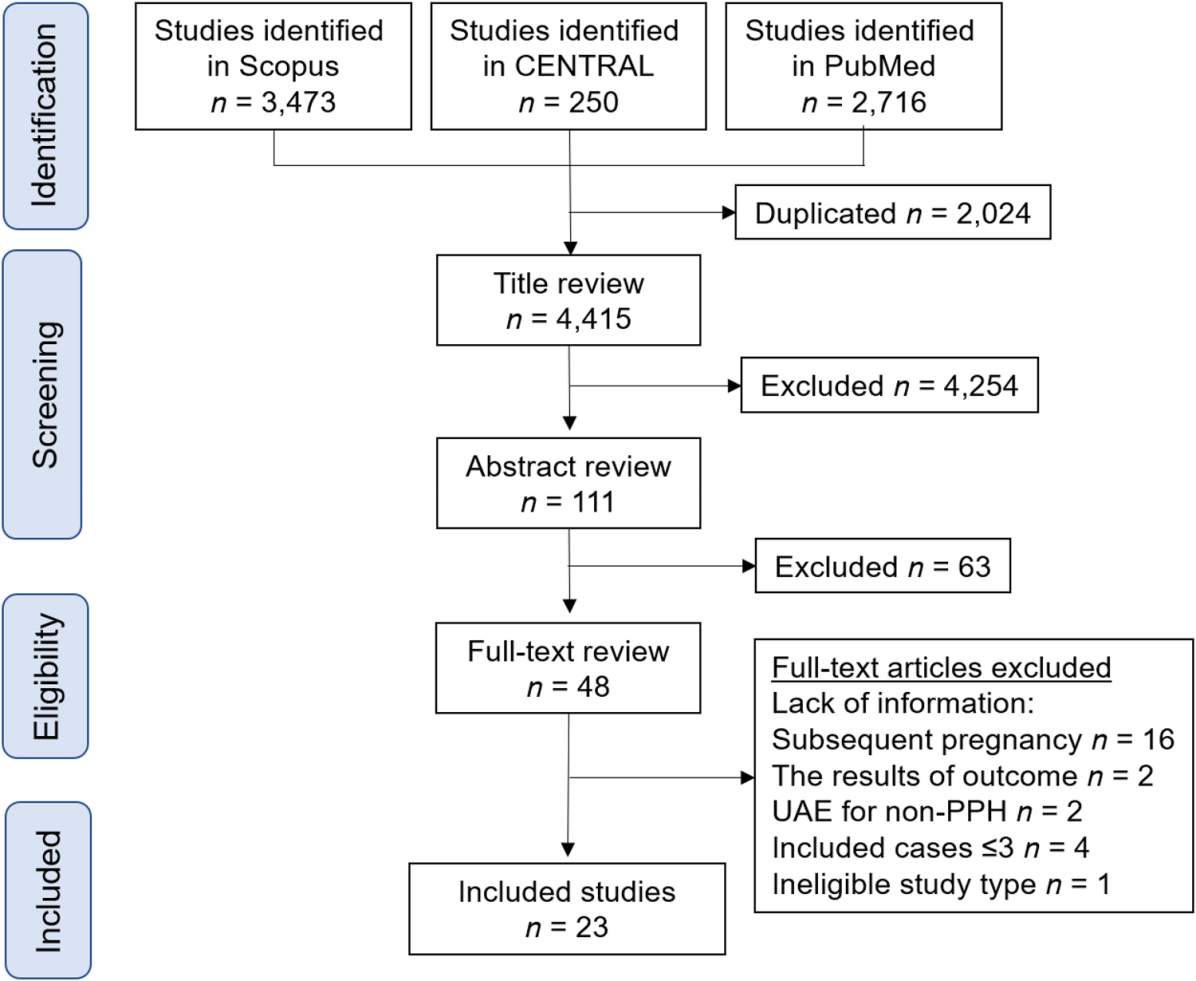
Study selection scheme used for the systematic review of the literature. *PPH* postpartum hemorrhage, *UAE* uterine artery embolization, *CENTRAL* Cochrane Central Register of Controlled Trials.

Figure 1 illustrates the study selection scheme. Overall, 6,439 studies were examined; among these, 23 studies^13,16,18–21,33–49^ including 483 pregnancies with prior UAE and 320,703 pregnancies without prior UAE met the inclusion criteria for the descriptive analysis.

#### Study characteristics

Supplemental Tables S3 and S4 summarize the metadata of the evaluated studies. Of the 23 included studies, 18 were non-comparator studies^13,16,21,34–48^ and five were comparator studies^18–20,33,49^. Among these five comparator studies, the patient’s background was matched by including only women with previous PPH in two studies^19,20^. In these two studies^19,20^, we determined the prevalence of obstetric complications for women with previous PPH with or without UAE. In another study, cases were matched in a 1:3 ratio by maternal age, parity, ethnicity, year and mode of delivery, birth weight, and gestational age^33^. The two remaining studies did not match the patients' backgrounds; therefore all pregnancies were included and divided into prior UAE and non-UAE groups^18,49^. Of the 18 non-comparator studies, one was a population-based observational study and the others were single-institution or multi-institution retrospective studies.

The included studies were published between 2003 and 2021. Nearly half of them were from Europe (*n* = 11, 47.8%), followed by Japan (*n* = 6, 26.1%), Korea (*n* = 3, 13.0%), the United States (*n* = 2, 8.7%), and Taiwan (*n* = 1, 4.3%). No studies examined the effect of prior UAE on the risk of PAS with a matched obstetric background.

#### Risk of bias in the included studies

Among the 23 studies, which were all retrospective, five had a non-randomized comparative design. No prospective studies were identified. The risk of bias assessment for the comparative studies demonstrated a possible moderate publication bias (moderate quality) in two studies^19,20^ and severe publication bias (low quality) in the other three studies (Supplemental Table S5)^18,33,49^.

#### Definitions of PAS and PPH

Among the 23 studies, six defined PAS. Specifically, four studies defined PAS as histopathologically confirmed PAS and two defined PAS as histopathologically and clinically confirmed PAS. Ten studies defined PPH based on the following transfusion requirements at delivery: > 2000 mL (one study), > 1000 mL (one study), > 1000 mL (cesarean delivery) (two studies) or > 500 mL (vaginal delivery) (one study), and > 500 mL (five studies).

### Meta-analysis

#### Risk of PAS and hysterectomy

Seventeen studies examined the rate of PAS after UAE for PPH during subsequent pregnancies, and 19 studies determined the rate of hysterectomy. The cumulative results of all studies indicated that the rates of PAS and hysterectomy were 16.3% (34/208) and 6.5% (28/432), respectively (Table 1).

**Table 1:** Summary of the rate of PAS and hysterectomy in subsequent pregnancies after UAE.

Author	Year	No	Age	PAS	Hyst	Def_PAS
**Comparator study**						
Jitsumori^18^	2020	16	35 (4.3)	6 (37.5%)	6 (37.5%)^†^	Path
	Control	3139^a, b^	33.7 (5.4)	37 (1.2%)	55 (1.8%)	
Imafuku^19^	2020	14	30.5 (26–38)	7 (50%)	–	Path/clin
	Control	32^a,b^	32.0 (21–41)	1 (3.1%)	–	
Cho^49^	2017	217	31.1 (3.5)	–	11 (5.1%)	–
	Control	317,453^a,b^	32.5 (3.1)	–	204 (0.1%)	–
Poggi^20^	2015	17	30.5 (5.5)	4 (23.5%)	3 (17.6%)	Path
	Control	18^a^	29.0 (6.0)	0	0	
**Non-comparator study**						
Ono^34^	2020	6	–	0	0	–
Toguchi^35^	2020	10^b^	–	4 (40.0%)	–	–
Cheng^16^	2017	14	–	–	0	–
Inoue^21^	2014	30	–	5 (16.7%)	5 (16.7%)	Path
Takeda^36^	2014	8	–	0	0	–
Lee^37^	2013	13^b^	–	0	0	–
Hardeman^38^	2010	11^b^	–	–	0	–
Sentilhes^13^	2009	19^b^	–	2 (10.5%)	1 (5.3%)	Path/clin
Fiori^39^	2009	11^b^	33 (20–43)	0	0	–
Gaia^40^	2009	18	–	3 (16.7%)	0	–
Chauleur^41^	2008	16^b^	–	0	0	–
Eriksson^42^	2007	6^b^	–	–	0	–
Shim^43^	2006	6	–	0	0	–
Descargues^44^	2004	6	–	0	0	–
Salomon^45^	2003	4	34.5 (34–36)	2 (50.0%)	2 (50.0%)	Path
Ornan^46^	2003	6	–	0	0	–
Picone^47^	2003	8	–	1 (12.5%)	0	–

Group		No		PAS	Hyst	
**Effect of UAE on the rate of PAS and hysterectomy (all studies)**						
UAE	–	456	–	34/208 (16.3%)	28/432 (6.5%)	

The median (range) or mean (standard deviation) or number (percentage per column) is shown. ^a^Women without prior UAE. ^b^Some patients had multiple deliveries. Some values listed above might be slightly different from the original values, as estimated by the authors.

*UAE* uterine artery embolization, *No*. number of prior uterine embolization cases, *PAS* placenta accreta spectrum, *Hyst* hysterectomy, *Def_PAS* definition of placenta accreta spectrum, *Path* pathology, *clin* clinical diagnosis.

**Figure 2:**
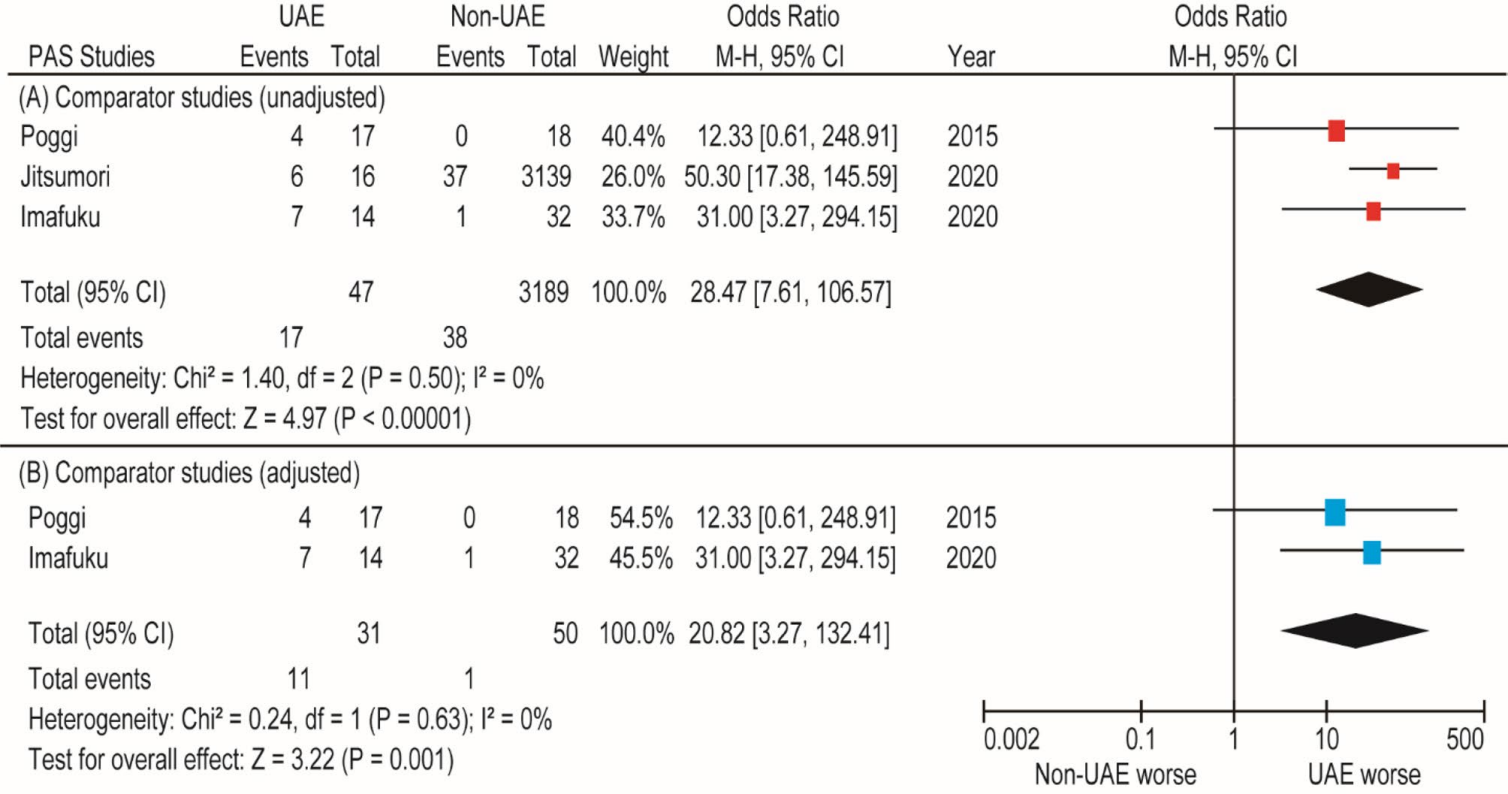
Results of the meta-analysis of the effect of prior UAE on the prevalence of PAS. The pooled odds ratios of (**A**) PAS and (**B**) PAS with previous PPH for women who did and did not undergo prior UAE. Some values listed might be slightly different from the original values because the calculation was performed using Revman ver. 5.4.1. *PAS* placenta accreta spectrum, *UAE* uterine artery embolization, *CI* confidence interval, *df* degrees of freedom.

We found three comparator studies that compared the rate of PAS between women who did and did not undergo prior UAE. In these three studies, 47 women underwent prior UAE and 3189 women did not undergo prior UAE. Considering the lack of heterogeneity, we conducted a fixed-effects analysis. The unadjusted pooled analysis (*n* = 3) demonstrated that women with prior UAE had a higher rate of PAS (OR 28.47, 95% CI 7.61–106.57) than those who did not undergo prior UAE (Fig. 2). In the adjusted pooled analysis (*n* = 2, all women had PPH during their previous delivery), prior UAE was associated with PAS (OR 20.82, 95% CI 3.27–132.41).

Twenty-one of 23 studies did not report the timing of the PAS diagnosis; therefore, the relationship between the timing of the PAS diagnosis and maternal outcomes could not be examined. Among the remaining two studies, the timing of the PAS diagnosis was mentioned for six women; all were diagnosed with PAS intrapartum. The rates of emergent cesarean deliveries and of multidisciplinary care and interventional radiology procedures were not reported.

**Figure 3:**
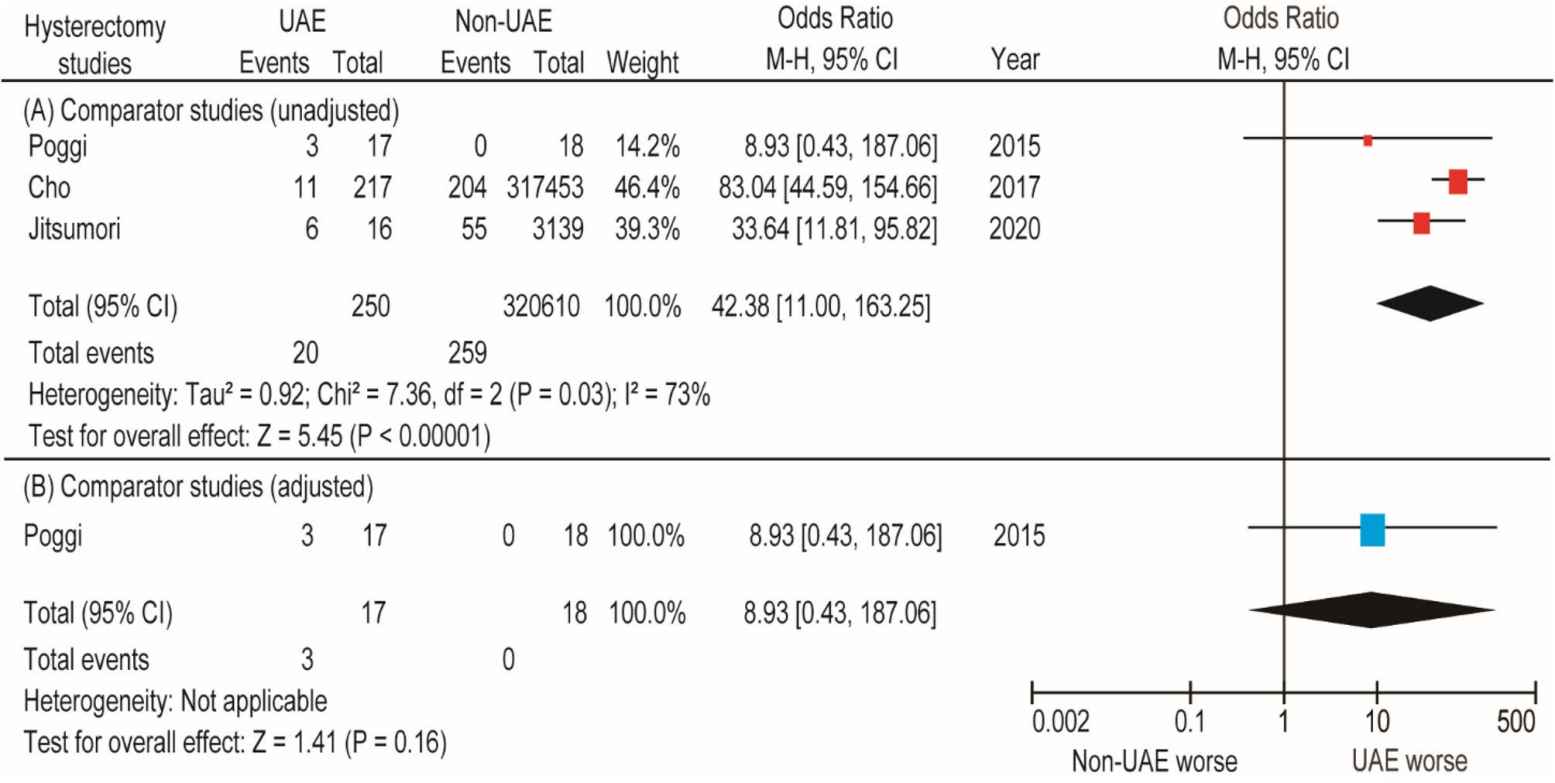
Results of the meta-analysis of the effect of prior UAE on the rate of hysterectomy. The pooled odds ratios of (**A**) hysterectomy and (**B**) hysterectomy with previous PPH for women who did and did not undergo prior UAE. Some values listed might be slightly different from the original values because the calculation was performed using Revman ver. 5.4.1. *UAE* uterine artery embolization, *CI* confidence interval, *df* degrees of freedom.

We also found three comparator studies that compared the rates of hysterectomy between women who did and did not undergo prior UAE. In these three studies, 250 women underwent prior UAE and 320,610 women did not undergo prior UAE. According to the unadjusted pooled analysis (*n* = 3), women who underwent prior UAE had a higher rate of hysterectomy (OR 42.38, 95% CI 11.00–163.25; heterogeneity: *P* = 0.03, *I*^2^ = 73%) than women who did not undergo prior UAE (Fig. 3). Because of the small number of studies examined, the risk of publication bias could not be calculated. According to the adjusted pooled analysis (*n* = 1), the rate of hysterectomy was higher for women who underwent prior UAE than for women who did not undergo prior UAE (17.6% [3/17] versus 0% [0/18]); however, the difference was not statistically significant (*P* = 0.16).

To examine maternal outcomes after delivery, rates of urinary tract injury and infection were reviewed. As shown in Supplemental Table S4, the rate of urinary tract injury was reported in one comparator study and three non-comparator studies. No urinary tract injuries were reported for women after UAE (*n* = 58). The rate of infection was not reported in the included studies.

#### Risk of PPH

**Figure 4:**
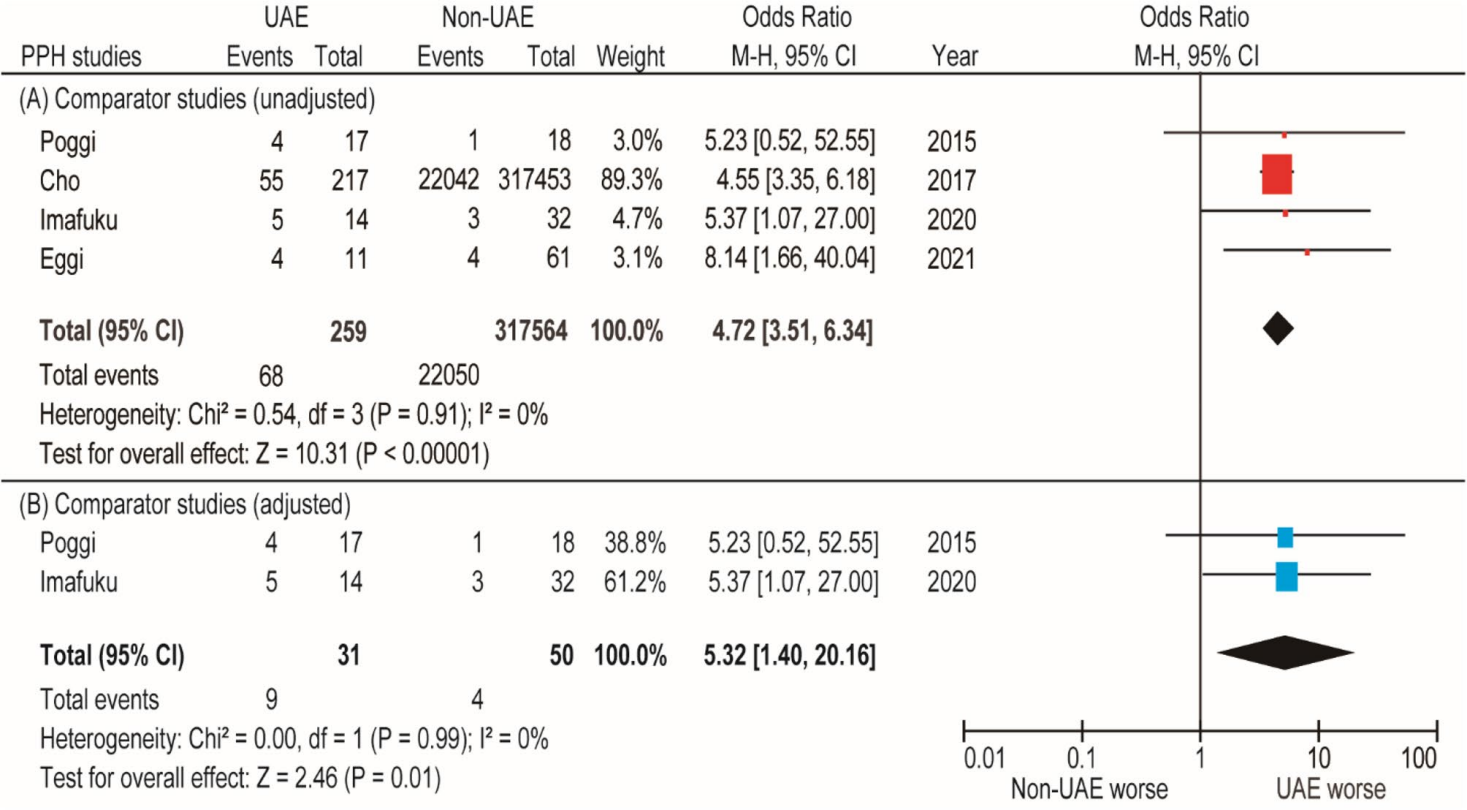
Results of the meta-analysis of the effect of prior UAE on the rate of PPH. The pooled odds ratio of (**A**) PPH and (**B**) PPH with previous PPH for women who did and did not undergo prior UAE. Some values listed might be slightly different from the original values because the calculation was performed using Revman ver. 5.4.1. *PPH* postpartum hemorrhage, *UAE* uterine artery embolization, *CI* confidence interval, *df* degrees of freedom.

Four comparator studies (three with low quality and one with moderate quality) examined the effect of prior UAE on the rate of PPH during subsequent pregnancies, and 19 non-comparator studies reported the rate of PPH for women who underwent prior UAE (Table 2). Because of the lack of heterogeneity, a fixed-effects analysis was performed. We found three comparator studies investigating 259 women who underwent prior UAE and 317,564 women who did not undergo prior UAE. According to the unadjusted pooled analysis (*n* = 4), women who underwent prior UAE were more likely to have PPH (OR 4.72, 95% CI 3.51–6.34; *P* < 0.01; heterogeneity: *P* = 0.91 and *I*^2^ = 0%) than women who did not undergo prior UAE (Fig. 4). However, the risk of publication bias could not be calculated because of the small number of included studies.

**Table 2:** Summary of the rate of PPH in subsequent pregnancies after UAE.

Author	Year	No	Age	PPH	UAE	Def_PPH
**Comparator study**						
Eggi^33^	2021	11	–	4 (36.4%)	–	> 500 ml
	Control	61	–	4 (6.6%)	–	
Imafuku^19^	2020	14	30.5 (26–38)	5 (35.7%)	–	> 2000 ml
	Control	32^a,c^	32.0 (21–41)	3 (9.4%)	–	
Cho^49^	2017	217	31.1 (3.5)	55 (25.3%)	13 (6.0)	–
	Control	317,453^a,c^	32.5 (3.1)	22,042 (6.9%)	328 (0.1)	–
Poggi^20^	2015	17	30.5 (5.5)	4 (23.5%)	1 (5.9%)	Transfusion
	Control	18^a^	29.0 (6.0)	1 (5.6%)	0	
**Non-comparator study**						
Grönvall^48^	2021	16^c,d^	–	3 (23.1%)^d^	–	–
Jitsumori^18^	2020	16	–	9 (56.3%)^b^	0	> 1000 ml
Ono^34^	2020	6	–	0	0	–
Toguchi^35^	2020	10^c^	–	1 (10.0%)	–	–
Cheng^16^	2017	14	–	2 (14.3%)^‡^	1 (7.1%)	> 500 ml
Inoue^21^	2014	8	–	0	0	> 500 ml (VD) > 1000 ml (CD)
Takeda^36^	2013	13^c^	–	0	0	–
Lee^37^	2014	30	–	7 (23.3%)	0	> 500 ml
Hardeman^38^	2010	11^c^		2 (18.2%)	0	–
Sentilhes^13^	2009	19^c^	–	6 (31.6%)	1 (5.3%)	–
Fiori^39^	2009	11^c^	33 (20–43)	1 (9.1%)	0	> 1000 ml
Gaia^40^	2009	18	–	3 (16.7%)	3 (16.7%)	> 500 ml
Chauleur^41^	2008	16^c^	–	1 (6.3%)	0	> 500 ml
Eriksson^42^	2007	6^c^		0	0	–
Shim^43^	2006	6	–	1 (16.7%)	0	–
Descargues^44^	2004	6	–	0	0	–
Salomon^45^	2003	4	34.5 (34–36)	4 (100%)	0	–
Ornan^46^	2003	6	–	0	0	–
Picone^47^	2003	8	–	7 (87.5%)	1 (12.5%)	–

Group		No	Age	PPH	UAE	
**Effect of UAE on the rate of PPH (all studies)**						
UAE	–	483	–	115/480 (24.0%)	20/432 (4.6%)	

Median (range) or mean (standard deviation) or number (percentage per column) are shown.

*UAE* uterine artery embolization, *VD* vaginal delivery, *CD* cesarean delivery, *No*. number of prior uterine embolization cases, *Indi* indication, *PPH* postpartum hemorrhage, *Def_PPH* definition of postpartum hemorrhage, *Type* type of uterine artery embolization.

^a^Women without prior UAE. ^b^Unpublished data. ^c^Some patients had multiple deliveries. ^d^PPH recurred in three of the 13 women (23.1%). Some values listed above might be slightly different from the original ones due to the estimation procedure used by the authors.

In the patient background-matched comparator analysis (all cases had prior PPH), the prevalence rates of recurrent PPH for women who did and did not undergo prior UAE were examined using a fixed analysis (*n* = 2). Women who underwent prior UAE were more likely to have recurrent PPH (OR 5.32, 95% CI 1.40–20.16, *P* < 0.01; heterogeneity: *P* = 0.99 and *I*^2^ = 0%) than women who did not undergo prior UAE (Fig. 4).

To estimate the severity of PPH, the rates of fresh-frozen plasma (FFP) and platelet transfusions and the prevalence of disseminated intravascular coagulation were explored. However, most studies did not report these data (Supplemental Table S4).

Two comparator studies reported the rates of UAE treatment. In one study (in which the patient background was not matched), the women who were treated with UAE during a previous pregnancy were more likely to have UAE than women in the control group (6.0% [13/217] versus 0.1% [328/317,453], *P* < 0.01)^48^. In another study (the patient background was matched with previous PPH), the rate of UAE for women treated with UAE during a previous pregnancy was similar to that of the control group (5.9% [1/17] versus 0% [0/18]; *P* = 0.47)^20^.

#### Risks of placenta previa, FGR, and PTB

To investigate the effect of prior UAE on placenta previa, FGR, and PTB during subsequent pregnancies, the individual rates of these complications were examined in four, two, and three comparator studies, respectively (Table 3). Furthermore, 11, 12, and 16 non-comparator studies reported the rates of placenta previa, FGR, and PTB, respectively.

**Table 3:** Summary of the rate of PP, FGR and PTB in subsequent pregnancies after UAE.

Author	Year	No	Age	PP	FGR	PTB
**Comparator study**						
Jitsumori^18^	2020	16	35 (4.3)	2 (12.5%)	1 (6.3%)	2 (12.5%)
	Control	3139^a,b^	33.7 (5.4)	123 (3.9%)	313 (10.0%)	446 (14.2%)
Imafuku^19^	2020	14	30.5 (26–38)	1 (7.1%)	1 (7.1%)	3 (21.4%)
	Control	32^a,b^	32.0 (21–41)	1 (3.1%)	0	3 (9.4)
Cho^49^	2017	217	31.1 (3.5)	20 (9.2%)	–	–
	Control	317,453^a,b^	32.5 (3.1)	2070 (0.7%)	–	–
Poggi^20^	2015	17	30.5 (5.5)	2 (11.8%)	–	3 (17.6%)
	Control	18^a^	29.0 (6.0)	1 (5.6%)	–	1 (5.6%)
**Non-comparator study**						
Grönvall^48^	2021	16^b^	–	–	0	0
Ono^34^	2020	6	–	0	0	0
Toguchi^35^	2020	10^b^	–	0	–	–
Cheng^16^	2017	14	–	–	1 (7.1%)	3 (21.4%)
Inoue^21^	2014	30	–	–	–	4 (13.3%)
Takeda^36^	2014	8	–	–	–	0
Lee^37^	2013	13^b^	–	–	–	2 (15.4%)
Hardeman^38^	2010	11^b^	–	0	1 (9.1%)	–
Sentilhes^13^	2009	19^b^	–	1 (5.3%)	0	0
Fiori^39^	2009	11^b^	33 (20–43)	0	0	1 (9.1%)
Gaia^40^	2009	18	–	0	–	0
Chauleur^41^	2008	16^b^	–	0	1 (6.3%)	1 (6.3%)
Eriksson^42^	2007	6^b^	–	–	–	2 (33.3%)
Shim^43^	2006	6	–	0	0	0
Descargues^44^	2004	6	–	0	0	0
Salomon^45^	2003	4	34.5 (34–36)	0	0	0
Ornan^46^	2003	6	–	0	0	0
Picone^47^	2003	8	–	–	0	2 (25.0%)

Group	–	No	–	PP	FGR	PTB
**Effect of UAE on the rate of complication**						
UAE	–	472	–	26/377(6.9%)	5/153 (3.3%)	23/234 (9.8%)

Median (range) or mean (standard deviation) or number (percentage per column) are shown. ^a^Women without prior UAE. ^b^ Some patients had multiple deliveries. Some values listed above might be slightly different from the original ones due to the estimation procedure used by the authors.

– not applicable, *PP* placenta previa, *FGR* fetal growth restriction, *PTB* preterm birth, *UAE* uterine artery embolization, *No*. number of prior uterine embolization cases.

The analysis that included both comparator and non-comparator studies indicated that the rates of placenta previa, FGR, and PTB were 6.9% (26/377), 3.3% (5/153), and 9.8% (23/234), respectively. According to the unadjusted pooled analysis, women who underwent prior UAE had higher rates of placenta previa (*n* = 3; OR 5.62, 95% CI 1.48–21.34; heterogeneity: *P* = 0.04 and *I*^2^ = 65%) (Supplemental Figure S1) than women who did not undergo UAE. Furthermore, the rates of FGR (*n* = 3; OR 1.48, 95% CI 0.14–15.39; heterogeneity: *P* = 0.20 and *I*^2^ = 38%) (Supplemental Figure S2) and PTB (*n* = 3; OR 1.63, 95% CI 0.64–4.17; heterogeneity: *P* = 0.49 and *I*^2^ = 0%) (Supplemental Figure S3) were similar between the two groups. There were few included studies; hence, the risk of publication bias could not be calculated.

According to the patient background-matched pooled analysis, women who underwent prior UAE did not have higher rates of placenta previa (OR 2.31, 95% CI 0.35–15.22), FGR (OR 7.22, 95% CI 0.28–188.69), and PTB (OR 3.00, 95% CI 0.74–12.14) than women who did not undergo prior UAE.

#### Embolic agents, particle sizes, and quantity of materials

The effects of embolic agents and the corresponding particle size on obstetric outcomes were examined. Among the 23 included studies, nine reported the embolic agents used. Most women with prior UAE were treated with a gelatin sponge. To estimate the particle size, the preparation of embolic agents was examined. Of 23 studies, five reported the following preparations of embolic agents: pumping (two studies); slurry (one study); cube (one study); and cutting (one study). No studies specified the quantity of materials used to treat PPH.

Since most cases were treated with a gelatin sponge, and because the information regarding agent preparation and the quantity of materials was limited, we did not investigate the association between obstetric outcomes and embolic agents.

## Discussion

### Key findings

There were two key findings during this study. First, prior UAE is a significant risk factor for PAS during subsequent pregnancies. Second, women who underwent prior UAE had a higher rate of PPH during subsequent pregnancies than women who did not undergo prior UAE; however, they did not have higher rates of other obstetric complications such as placenta previa, FGR, and PTB.

### Comparison with existing literature

Of the conditions associated with PPH, PAS has the highest risk. Furthermore, PAS is associated with increased maternal morbidity and mortality rates caused by massive hemorrhage during delivery^50–54^. For women with PAS, the mean blood loss during cesarean delivery is approximately 3000 mL, and the hysterectomy rate is approximately 40–70%^50–54^. The main risk factor for PAS is placenta previa, with an approximate OR of 50–100^55–57^. However, PAS has been linked to other risk factors, including a history of cesarean delivery (OR 5–9), uterine surgery (OR 2–3), multiparity (OR 3), advanced maternal age (OR 2.1)^56,58^, and in vitro fertilization embryo transfer (OR 3–14)^59–63^. Although the rate of PAS could be high for women who underwent prior UAE for PPH, this has not been determined by meta-analyses.

During our study, the OR of prior UAE was 28.47 in the unadjusted analysis and 20.82 in the adjusted analysis. Therefore, prior UAE may be a substantial risk factor for PAS. Since women with PAS often need to undergo hysterectomy because of severe PPH, the high rate of hysterectomy may have been caused by the increasing rate of PAS^50,51^.

Our study had several possible biases. As shown in Table 4, the estimated recurrence rates of PAS^64–67^, placenta previa^64,68,69^, PPH^70,71^, FGR^72,73^, and PTB^74,75^ have been widely reported as high during subsequent pregnancy. For instance, nearly half of PAS cases involve PPH, and homeostasis is often achieved by UAE^64–67,76,77^. Therefore, it should be noted that our study and previous studies could not exclude the effect of the presence of PAS during previous pregnancies. A previous report indicated that the rate of recurrent PAS (including clinical PAS) was 19.9%^65^. Therefore, if PPH is caused by PAS during the first pregnancy, then the risk of PAS during the subsequent pregnancy may be high. Similarly, women who had PPH, FGR, and PTB during the previous pregnancy had a high rate of recurrence of these complications (Table 4). Therefore, a patient background-matched study with a larger sample size is warranted to examine the effect of PAS on obstetric complications.

**Table 4:** The estimated prevalence of obstetric complications and the recurrence rate of each complication.

Disease	Prevalence (%)	Recurrence rate (%)
PAS^64–66^	0.1–3^a^	~ 20.0
Hysterectomy^78^	0.10	–
PPH^70,71^	1–5	20–25
Placenta previa^64,68,69^	0.3–1.0	2–8
FGR^72,73^	4–6	20–25
PTB^74,75^	4–6	20–30

*PAS* placenta accreta spectrum, *PPH* postpartum hemorrhage, *FGR* fetal growth restriction, *PTB* preterm birth.

^a^Including pathological and clinical diagnosis of placenta accreta spectrum.

An antenatal diagnosis of PAS helps reduce hemorrhagic morbidity and improves the prognosis, possibly because of the comprehensive multidisciplinary care received by patients, which includes planned cesarean hysterectomy, transfusion preparation, and treatment administered by skilled physicians^79–82^. Therefore, the timing of diagnosis of PAS is an important factor to examine, especially because undiagnosed PAS is associated with adverse maternal outcomes^22,79,83^. Moreover, while multidisciplinary care and/or interventional radiology procedures have the potential to improve the maternal outcomes of PAS, these data were not available; this lack of data was a limitation of this study^23,51^.

Although limited, the available data (*n* = 6) indicated that all women with PAS after prior UAE were diagnosed intrapartum. We believe that an understanding of the effect of prior UAE on the risks of PAS and hysterectomy during subsequent pregnancies would be helpful to making an antenatal diagnosis of PAS. Notably, prior UAE may be a strong risk factor for PAS, and women who underwent prior UAE may have PAS even without placenta previa.

A recent study that compared PAS in women without placenta previa (*n* = 106) and PAS in women with placenta previa (*n* = 245) revealed that PAS without placenta previa is less likely to be diagnosed antepartum (OR 0.1, 95% CI 0.05–016)^50^. Considering that placenta previa is a significant risk factor for PAS, antepartum evaluations might be performed more carefully for women with placenta previa than for women without placenta previa. This step may lead to a lower diagnosis rate for women without placenta previa^50^. Additionally, a low diagnosis rate can potentially lead to the missed opportunity for multidisciplinary team management (OR 0.11, 95% CI 0.07–019)^50^. Despite the absence of placenta previa and less placental invasion, severe maternal morbidity during delivery was similar between groups (18.9% versus 19.6%, OR 0.88, 95% CI 0.49–1.59)^50^.

The current study results demonstrated that knowledge of prior UAE as a high-risk factor for PAS, PPH, and high hysterectomy rates are useful for clinicians. Additionally, pregnant women with prior UAE need to undergo careful antepartum evaluations for PAS and prepare for PPH during delivery.

Some studies have discussed the relationship between prior UAE and a high rate of PAS. Uterine necrosis is a complication of UAE performed for PPH, and reduced blood flow to the uterus and damage to the endometrium may lead to uterine necrosis^84^. We hypothesized that endometrial damage occurs even in women without complications who are treated with UAE. Endometrial damage is a risk factor for PPH and may be one of the causes of PAS.

Previous studies have suggested that embolic agents, particle sizes, and the quantity of materials could potentially affect the short-term or long-term complications of UAE, including endometrial damage. A previous animal study involving renal artery embolization was performed with different embolic agents for dogs and examined recanalization of embolic vessels according to the type of embolic agent^85^. Complete recanalization was observed with an absorbable gelatin sponge and no recanalization was observed with non-absorbable materials. Cases of uterine necrosis after UAE involving non-absorbable materials have been reported as well^86,87^.

Although a gelatin sponge is an absorbable embolic agent, the smaller size of the gelatin sponge is associated with a higher rate of complications such as uterine necrosis and intrauterine synechia^40,84,88^. Therefore, the association between the complication rate and the size of the embolic agent for UAE is relevant. However, a clinical study that compared the incidence of complications based on the size of embolic agent for UAE was underpowered^88^. Moreover, this study revealed that the effect of the size of embolic agent for UAE on obstetric outcomes during subsequent pregnancy is unclear.

Large volumes of embolic agents for UAE or repeated UAE for severe PPH may be associated with higher complication rates^89–91^. A possible reason for this association is that large-volume embolic agents may simultaneously block the upper and lower anastomotic uterine blood supplies, thus leading to a high probability of necrosis or damage of the uterus^92^.

We hypothesized that non-absorbable embolic agents, small particles, and the use of large volumes of embolic agents could potentially increase the rate of long-term complications and worsen the obstetric outcomes during a subsequent pregnancy. However, we could not examine the effects of these factors on obstetric outcomes; this was another limitation of this study. Further studies should examine the effects of type, size, and quantity of embolic agents used during a prior UAE on the obstetric outcomes during subsequent pregnancies.

### Strengths and limitations

One strength of this study is that it is likely the first systematic review to focus on the effect of prior UAE for PPH on PAS during subsequent pregnancies. Our study revealed that prior UAE performed to treat PPH is associated with high rates of PAS and PPH. However, as mentioned, this study had additional limitations. First, bias was not measured because all the included studies were retrospective. Potential sources of confounding variables in the study included the varying definitions of PAS and PPH across studies, unmatched patient backgrounds, and the lack of data regarding UAE indications during previous pregnancies. In particular, different definitions of PAS and PPH among studies may have caused severe bias; thus, we should note this as a strong limitation of this study. Another important limitation was that no studies matched the obstetric patient backgrounds to examine the effect of prior UAE for PPH on the rate of obstetric complications. Second, we only found two comparator studies that examined the rate of obstetric complications of women with previous PPH who did and did not undergo prior UAE. Because PPH and its causes (e.g., PAS and placenta previa) are highly recurrent, future studies of background-matched patient cohorts are necessary. Third, the embolic agent used for prior UAE, the severity of PPH, the timing of the PAS diagnosis, the transfusion rates (FFP and platelet), the prevalence of disseminated intravascular coagulation, and the presence of PAS were not identified in most studies. These factors might have influenced the results of this study; hence, the lack of such data is a notable limitation of this study. Fourth, publication bias is a matter of concern because the negative relationship between prior UAE for PPH and PAS might not have been reported in the original articles. To confirm the results of this study, a more robust study should be conducted. Considering that a randomized control study is difficult to conduct because of the rarity of women who underwent prior UAE for PPH, a prospective study seems appropriate. Fifth, the sample size was limited in most studies; thus, the possibility of type II error needs to be recognized, especially in the interpretation of the results of the adjusted pooled analysis. Sixth, the type of embolic agent, particle size, and quantity of agents may have affected the obstetric outcomes of women with prior UAE. However, only limited data were available in our included studies; therefore, we could not examine such associations. Further investigations are warranted to improve obstetric outcomes after UAE. Finally, the protocol of the systematic review has not been registered. Without preregistration, it is unknown whether the main outcomes, such as PAS, were predefined as primary outcomes. Therefore, this could cause bias of the systematic review and should be noted as a limitation of this study.

### Conclusions

Prior UAE for PPH may be a significant risk factor for PAS. Moreover, PPH frequently recurs. Therefore, we should note that pregnant women who underwent prior UAE are at high risk for PPH during subsequent pregnancies. To confirm the results of this study, a patient background-matched study or prospective study exploring the effect of prior UAE is warranted.

## Supplementary Information﻿

Supplementary Information 1.

## Data Availability

All the studies used in this study are published in the literature.
