# Supplementary material for: A systematic review and meta-analysis of obstetric and maternal outcomes after prior uterine artery embolization
Source: Sci Rep. 2021 Aug 19;11:16914. doi: 10.1038/s41598-021-96273-z (PMC8377070; doi:10.1038/s41598-021-96273-z)
Supplement: Supplementary file 1 — Supplementary Information 1. [file 41598_2021_96273_MOESM1_ESM.docx]

**Supplemental Figure S1. Results of the meta-analysis for the effect of prior UAE on placenta previa prevalence.**


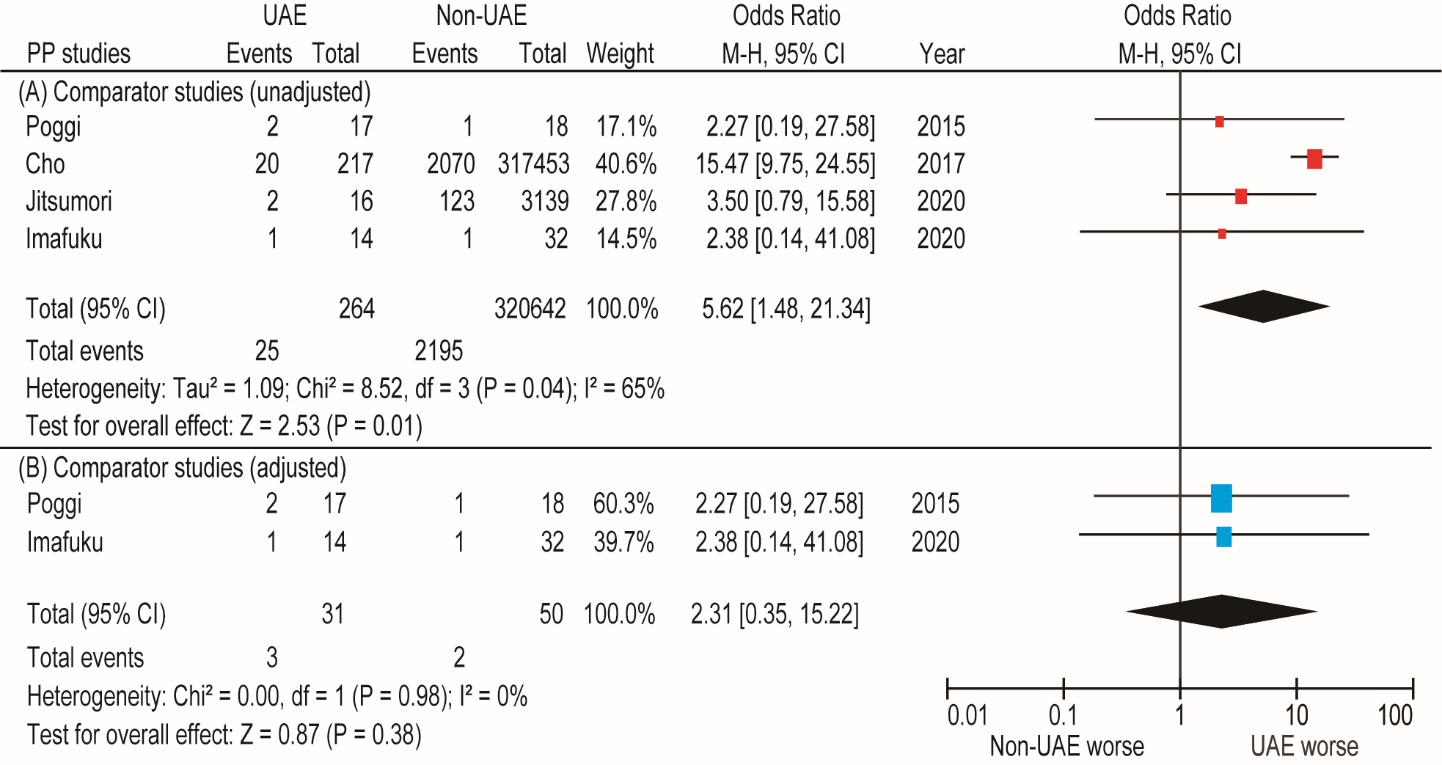


The pooled odds ratio for (A) placenta previa (unadjusted) and (B) placenta previa (patient background-matched with previous PPH) between women who did and did not undergo prior UAE is shown. Some values listed above might be slightly different from the original values because of the calculation in Revman ver. 5.4.1. Abbreviations: PP, placenta previa; UAE, uterine artery embolization; CI, confidence interval and ; df, degrees of freedom.

**Supplemental Figure S2. Results of the meta-analysis for the effect of prior UAE on the rate of fetal growth restriction.**


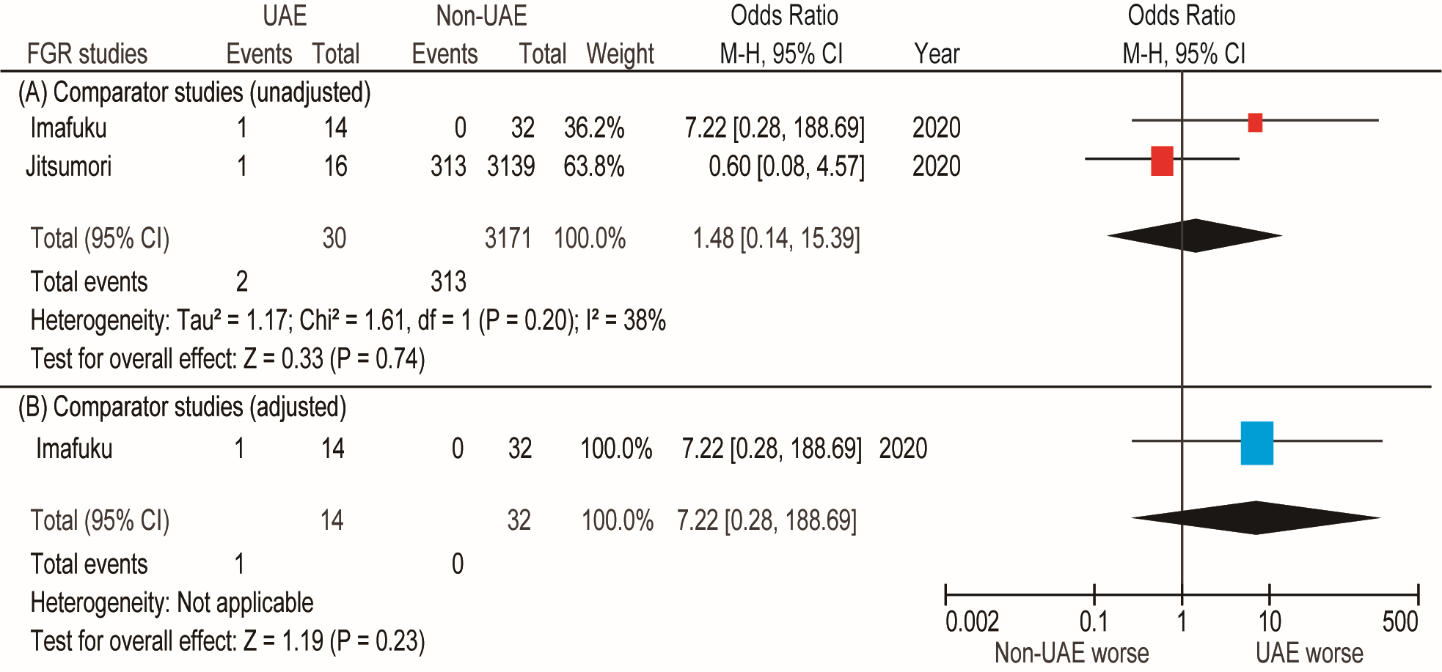


The pooled odds ratio for (A) fetal growth restriction (unadjusted) and (B) fetal growth restriction (patient background-matched with previous PPH) between women who did and did not undergo prior UAE is shown. Some values listed above might be slightly different from the original values because of the calculation in Revman ver. 5.4.1. Abbreviations: FGR, fetal growth restriction; UAE, uterine artery embolization; CI, confidence interval and ; df, degrees of freedom.

**Supplemental Figure S3. Results of the meta-analysis for the effect of prior UAE on the rate of preterm birth.**


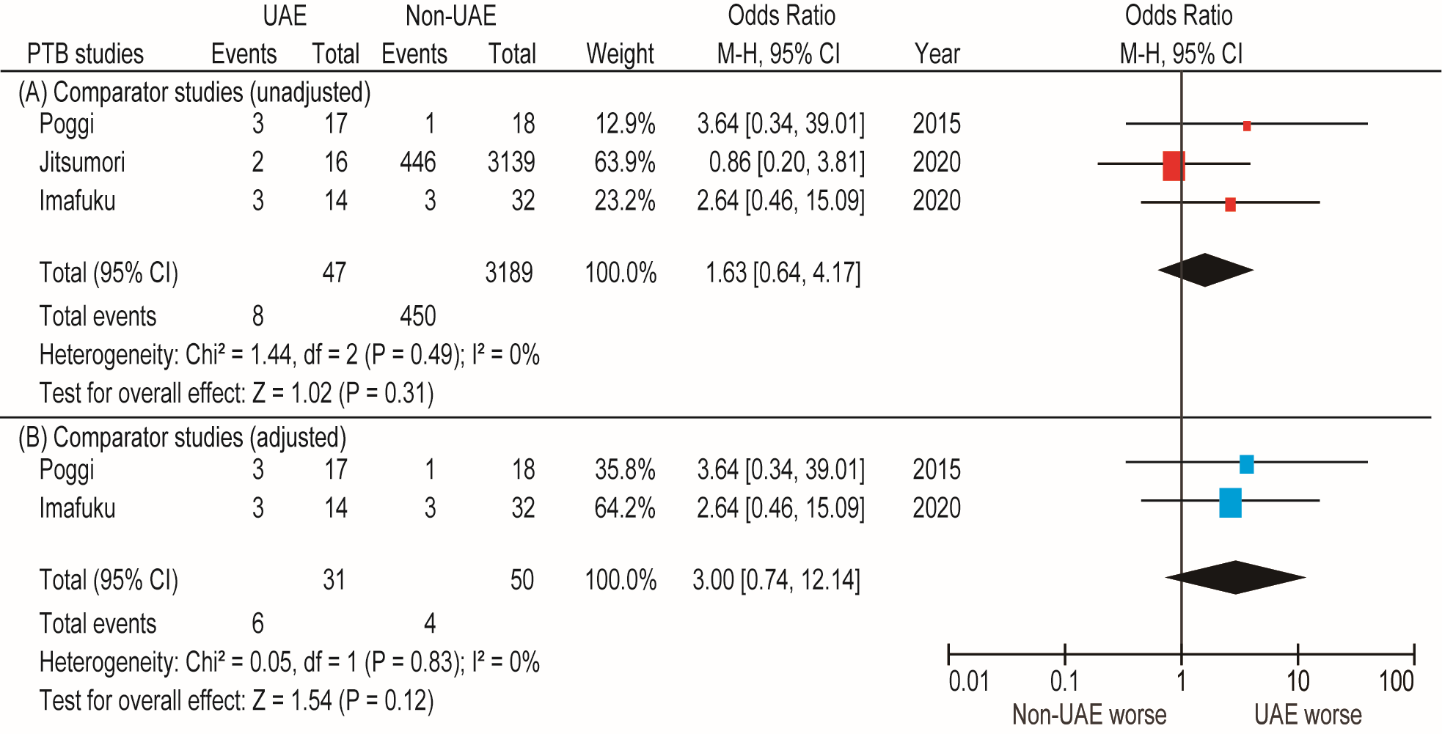


The pooled odds ratio for (A) preterm birth (unadjusted) and (B) preterm birth (patient background-matched with previous PPH) between women who did and did not undergo prior UAE is shown. Some values listed above might be slightly different from the original values because of the calculation in Revman ver. 5.4.1. Abbreviations: PTB, preterm birth; UAE, uterine artery embolization; CI, confidence interval and ; df, degrees of freedom.

**Supplemental Table S1. The search strategy.**

PubMed

(Embolization, Therapeutic[Mesh:NoExp] OR Uterine Artery Embolization[MeSH] OR UAE[TIAB] OR Interventional-radiology[TIAB] OR pelvic-embo*[TIAB] OR artery-embo*[TIAB] OR arterial-embo*[TIAB] OR TAE[TIAB] OR angiographic-embo*[TIAB] OR transarterial-embo*[TIAB]) AND (Pregnancy [MeSH] OR Birth intervals[MeSH] OR Fertility[MeSH] OR pregnanc*[TIAB] OR pregnant[TIAB] OR gravid*[TIAB] OR obstet*[TIAB] OR postpartum*[TIAB] OR birth[TIAB] OR fetus*[TIAB] OR foetus*[TIAB] OR fetal[TIAB] OR foetal[TIAB] OR gestation[TIAB] OR gestations[TIAB]) 2,716

Cochrane Central Register of Controlled Trials#1 ((transart* or arterial or artery or angiographic or pelvic or obstet*) NEAR/2 (embolizat* or embolisat*)):ti,ab,kw 780

#2 UAE:ti,ab,kw OR TAE:ti,ab,kw OR interventional radiology:ti,ab,kw 3,382

#3 MeSH descriptor: [Uterine Artery Embolization] explode all trees 352

#4 MeSH descriptor: [Embolization, Therapeutic] this term only 52

#5 #1 OR #2 OR #3 OR #4 4,157

#6 gravid*:ab,ti,kw OR obstet*:ab,ti,kw OR postpartum*:ab,ti,kw OR birth:ab,ti,kw OR fetus:ab,ti,kw OR foetus:ab,ti,kw OR fetal:ab,ti,kw OR foetal:ab,ti,kw OR gestation:ab,ti,kw OR gestations:ab,ti,kw 56,998

#7 MeSH descriptor: [Pregnancy] explode all trees 21,594

#8 pregnanc*:ab,ti,kw OR pregnant:ab,ti,kw 66,055

#9 MeSH descriptor: [Birth intervals] explode all trees 18

#10 MeSH descriptor: [Fertility] explode all trees 1

#11 #6 OR #7 OR #8 OR #9 OR #10 91,552

#12 #5 AND #11 250

Scopus

#1 TITLE-ABS-KEY (transart* or arterial or artery or angiographic or pelvic or obstet*) W/2 (embolizat* or embolisat*) 21,257

#2 TITLE-ABS-KEY (UAE OR "Interventional radiology" OR TAE) 43,010

#3 #1 OR #2 60,221

#4 TITLE-ABS-KEY (gravid*" OR "obstet*" OR "postpartum* OR birth OR fetus* OR foetus* OR fetal OR foetal OR gestation OR gestations) 1,242,163

#5 TITLE-ABS-KEY (pregnanc* OR pregnant) 1,146,177

#6 #4 OR #5 1,788,235

#7 #3 AND #6 3,473

**Supplemental Table S2. PICOS criteria for inclusion of systematic review.**

| Population | Pregnant women |
| --- | --- |
| Intervention | Prior UAE (with or without previous PPH) |
| Comparison | No prior UAE |
| Outcome | Obstetric outcome (the rates of PAS, hysterectomy, PPH, placenta previa, FGR, PTB, and UAE)  Maternal outcome (the rate of transfusion [RBC, FFP, PLT], urinary tract injury, and infection) |
| Study design | Retrospective or prospective cohort studies, case-control study, and randomized controlled trials |

Abbreviations: PICOS, Patient/Population, Intervention, Comparator, Outcome, Study; UAE, uterine artery embolization; PPH, postpartum hemorrhage; PAS, placenta accreta spectrum; FGR, fetal growth restriction; PTB, preterm birth; IVF-ET, *in vitro* fertilization-embryo transfer; and ET, embryo transfer.

**Supplemental Table S3. Metadata of systematic review.**

| Author | Year | Area | No. | Cause | Def_PAS | Def_PPH | Timing of diagnosis | Embolic agents | Preparation |
| --- | --- | --- | --- | --- | --- | --- | --- | --- | --- |
| **Comparator study** |  |  |  |  |  |  |  |  |  |
| Eggel^1^ | 2021 | CHE | 11 | -- | -- | >500ml | -- | -- | -- |
|  |  | Control | 61 | -- | -- |  | -- | -- | -- |
| Jitsumori^2^ | 2020 | JPN | 16 | # | Path | >1000ml | -- | -- | -- |
|  |  | Control: | 3139^§^ |  |  |  | -- | -- | -- |
| Imafuku^3^ | 2020 | JPN | 14 | $ | Path/ clin | >2000ml | Intra: 4/7 (57.1%), Unk: 3/7 (42.9%) | Gelatin sponge: all | -- |
|  |  | Control | 32^§^ | & |  |  | -- | -- | -- |
| Cho^4^ | 2017 | KOR | 217 | -- | -- | -- | -- | -- | -- |
|  |  | Control: | 317,453^§^ | -- | -- | -- | -- | -- | -- |
| Poggi^5^ | 2015 | USA | 17 | ¶ | Path | Transfusion | -- | Gelatin sponge: 4, Unk: 13 | Slurry |
|  |  | Control: | 18 | \| |  |  | -- |  |  |
| **Non-comparator study** |  |  |  |  |  |  |  |  |  |
| Grönvall^6^ | 2021 | FIN | 16^§^ | -- | -- | -- | -- | -- | -- |
| Ono^7^ | 2020 | JPN | 6 | -- | -- | -- | -- | -- | -- |
| Toguchi^8^ | 2020 | JPN | 10^§^ | -- | -- | -- | -- | Gelatin sponge: all | Pumping |
| Cheng^9^ | 2017 | TWN | 14 | -- | -- | >500ml | -- | Gelatin sponge: all | Cube |
| Inoue^10^ | 2014 | JPN | 30 | -- | Path | >500ml | -- | -- | -- |
| Takeda^11^ | 2014 | JPN | 8 | -- | -- | >500ml (VD)  > 1000ml (CD) | -- | -- | -- |
| Lee^12^ | 2013 | KOR | 13^§^ | -- | -- | -- | -- | -- | -- |
| Hardeman^13^ | 2010 | FRN | 11^§^ | -- | -- | -- | -- | Gelatin sponge: 10, gelatin + coil: 1 | -- |
| Sentilhes^14^ | 2009 | FRN | 19^§^ | -- | Path/ clin | -- | Intra: 2/2 (100) | -- | -- |
| Fiori^15^ | 2009 | FRN | 11^§^ | -- | -- | >1000ml | -- | -- | -- |
| Gaia^16^ | 2009 | FRN | 18 | -- | -- | >500ml | -- | Gelatin sponge: all | -- |
| Chauleur^17^ | 2008 | FRN | 16^§^ | -- | -- | >500ml | -- | -- | -- |
| Eriksson^18^ | 2007 | SWE | 6^§^ | -- | -- | -- | -- | Gelatin sponge: all | Pumping^ |
| Shim^19^ | 2006 | KOR | 6 | -- | -- | -- | -- | -- | -- |
| Descargues^20^ | 2004 | FRN | 6 | -- | -- | -- | -- | -- | -- |
| Salomon^21^ | 2003 | FRN | 4 | -- | Path | -- | -- | Gelatin sponge: all | -- |
| Ornan^22^ | 2003 | USA | 6 | -- | -- | -- | -- | Gelatin sponge: all | Cutting |
| Picone^23^ | 2003 | FRN | 8 | -- | -- | -- | -- | -- | -- |

Number (percentage per column) is shown. # 9 patients: atonic bleeding, manual removal of placenta: 3 patients, low-lying placenta 1 patient, Unknown 1 patient, retained pregnancy of conception: 1 patient. $ Uterine atony: 10 patients, clinical placenta accreta spectrum: 2 patients, placenta previa or low-lying placenta: 2 patients. & Uterine atony: 16 patients, clinical placenta accreta spectrum: 1 patient, placenta previa or low-lying placenta: 9 patients, and others: 6 patients. ¶ Uterine atony: 10 patients, abruption: 1 patient, placenta previa: 1 patient, cervical or vaginal laceration: 3 patients, placenta accreta spectrum: 2 patients. |Uterine atony: 5 patients, abruption: 4 patients, placenta previa: 1 patient, cervical or vaginal laceration: 2 patients, uterine artery laceration: 1 patient, uterine rupture: 1 patient; retained products of concept: 2 patients, DIC with preeclampsia: 1 patient. ^ Approximately 1–3 cm^2^ of Gelfoam was suspended in a solution mixed in a 20-ml syringe with 10 ml saline and 10 ml contrast medium. ^§^ Some patients have multiple deliveries. Some values listed above might be slightly different form original values due to estimating by authors. Abbreviations: --, not applicable, UAE, uterine artery embolization, No., number of prior uterine embolization cases; cause, cause of postpartum hemorrhage in previous pregnancy; PAS, placenta accreta spectrum; PP, placenta previa; PP, placenta previa; PPH, postpartum hemorrhage; FGR, fetal growth restriction; PTB, Preterm birth; VD, vaginal delivery; CD, cesarean delivery; Hyst, hysterectomy; Intra, intrapartum diagnosis; Unk, unknown; preparation, the type of preparation of embolic agents; Def_PAS, definition of placenta accreta spectrum; Def_PPH, definition of postpartum hemorrhage; JPN, Japan; TWN, Taiwan; FRN, France; KOR, Korea; USA, United States of America; SWE, Sweden; Path, pathology; clin, clinical diagnosis.

**Supplemental Table 4. Summary of obstetric outcome of the meta-analysis.**

| Author | Year | No. | PAS | PP | PPH | FGR | PTB | UAE | Hyst | Urinary | Infection | Transfusion | FFP | PLT |
| --- | --- | --- | --- | --- | --- | --- | --- | --- | --- | --- | --- | --- | --- | --- |
| **Comparator study** |  |  |  |  |  |  |  |  |  |  |  |  |  |  |
| Eggel^1^ | 2021 | 11 | -- | -- | 4 (36.4%) | -- | 0 | -- | -- | -- | -- | -- | -- | -- |
|  |  | 61 | -- | -- | 4 (6.6%) | -- | -- | -- | -- | -- | -- | -- | -- | -- |
| Jitsumori^2^ | 2020 | 16 | 6 (37.5) | 2 (12.5) | 9 (56.3)^†^ | 1 (6.3) | 2 (12.5) | 0 | 6 (37.5)^†^ | -- | -- | -- | -- | -- |
|  |  | 3139^§^ | 37 (1.2) | 123 (3.9) | -- | 313 (9.9) | 446 (14.2) | -- | 55 (1.75) | -- | -- | -- | -- | -- |
| Imafuku^3^ | 2020 | 14 | 7 (50) | 1 (7.1) | 5 (35.7) | 1 (7.1) | 3 (21.4) | -- | -- | -- | -- | -- | -- | -- |
|  |  | 32^§^ | 1 (3.1) | 1 (3.1) | 3 (9.4) | 0 | 3 (9.4) | -- | -- | -- | -- | -- | -- | -- |
| Cho^4^ | 2017 | 217 | -- | 20 (9.2) | 55 (25.3) | -- | -- | 13(6.0) | 11 (5.1) | -- | -- | -- | -- | -- |
|  |  | 317,453^§^ | -- | 2070 (0.7) | 22,042 (6.9) | -- | -- | 328 (0.1) | 204 (0.1) | -- | -- | -- | -- | -- |
| Poggi^5^ | 2015 | 17 | 4 (23.5) | 2 (11.8) | 4 (23.5) | -- | 3 (17.6) | 1 (5.9) | 3 (17.6) | 0 | -- | 4 (23.5) | 1 (5.9) | 0 |
|  |  | 18 | 0 | 1 (5.6) | 1 (5.6) | -- | 1 (5.6) | 0 | 0 | 0 | -- | 1 (5.6) | 0 | 0 |
| **Non-comparator** study |  |  |  |  |  |  |  |  |  |  |  |  |  |  |
| Grönvall^6^ | 2021 | 16^§^ | -- | -- | 3 (23.1)* | 0 | 0 | -- | -- | -- | -- | -- | -- | -- |
| Ono^7^ | 2020 | 6 | 0 | 0 | 0 | 0 | 0 | 0 | 0 | -- | -- | -- | -- | -- |
| Toguchi^8^ | 2020 | 10^§^ | 4 (40) | 0 | 1 (10) | -- | -- | -- | -- | -- | -- | -- | -- | -- |
| Cheng^9^ | 2017 | 14 | -- | -- | 2 (18.2)^‡^ | 1 (9.1)^‡^ | 3 (21.4) | 1 (9.1) | 0 | -- | -- | -- | -- | -- |
| Inoue^10^ | 2014 | 30 | 5 (16.7) | -- | 7 (23.3) | -- | 4 (13.3) | 0 | 5 (16.7) | -- | -- | -- | -- | -- |
| Takeda^11^ | 2014 | 8 | 0 | -- | 0 | -- | 0 | 0 | 0 | -- | -- | -- | -- | -- |
| Lee^12^ | 2013 | 13^§^ | 0 | -- | 0 | -- | 2 (15.4) | 0 | 0 | -- | -- | -- | -- | -- |
| Hardeman^13^ | 2010 | 11^§^ | -- | 0 | 2 (18.2) | 1 (9.1) | -- | 0 | 0 | -- | -- | -- | -- | -- |
| Sentilhes^14^ | 2009 | 19^§^ | 2 (10.5) | 1 (5.3) | 6 (31.6) | 0 | 0 | 1 (5.3) | 1 (5.3) | 0 | -- | -- | -- | -- |
| Fiori^15^ | 2009 | 11^§^ | 0 | 0 | 1 (9.1) | 0 | 1 (9.1) | 0 | 0 | -- | -- | -- | -- | -- |
| Gaia^16^ | 2009 | 18 | 3 (16.7) | 0 | 3 (16.7) | -- | 0 | 3 (16.7) | 0 | -- | -- | -- | -- | -- |
| Chauleur^17^ | 2008 | 16^§^ | 0 | 0 | 1 (6.3) | 1 (6.3) | 1 (6.3) | 0 | 0 | 0 | -- | 0 | 0 | 0 |
| Eriksson^18^ | 2007 | 6^§^ | -- | -- | 0 | -- | 2 (33.3) | 0 | 0 | -- | -- | -- | -- | -- |
| Shim^19^ | 2006 | 6 | 0 | 0 | 1 (16.7) | 0 | 0 | 0 | 0 | -- | -- | -- | -- | -- |
| Descargues^20^ | 2004 | 6 | 0 | 0 | 0 | 0 | 0 | 0 | 0 | -- | -- | -- | -- | -- |
| Salomon^21^ | 2003 | 4 | 2 (50) | 0 | 4 (100) | 0 | 0 | 0 | 2 (50) | -- | -- | 2 (50) | -- | -- |
| Ornan^22^ | 2003 | 6 | 0 | 0 | 0 | 0 | 0 | 0 | 0 | 0 | -- | 0 | 0 | 0 |
| Picone^23^ | 2003 | 8 | 1 (12.5) | -- | 7 (87.5) | 0 | 2 (25) | 1 (12.5) | 0 | -- | -- | 2(25) | -- | -- |

Number (percentage per column) is shown. ^§^ Some patients have multiple deliveries. *PPH recurred in three of the 13 women (23.1%). ^‡^Women with preterm birth were excluded from the analysis. ^†^Unpublished data. Some values listed above might be slightly different form original values due to estimating by authors. Abbreviations: --, not applicable, UAE, uterine artery embolization, No., number of prior uterine embolization cases; cause, cause of postpartum hemorrhage in previous pregnancy; PAS, placenta accreta spectrum; PP, placenta previa; PP, placenta previa; PPH, postpartum hemorrhage; FGR, fetal growth restriction; PTB, Preterm birth; Hyst, hysterectomy; urinary, urinary tract injury; infection, infection after delivery; FFP, frozen fresh plasma; PLT, platelet transfusion.

**Supplemental Table S5. Risk of bias assessment for the comparator study.**

| Authors | Confounding | Selection | Classification of intervention | Deviations from interventions | Missing data | Measurement of outcomes | Reported results | Overall bias |
| --- | --- | --- | --- | --- | --- | --- | --- | --- |
| Eggel^1^ | ● | ● | ● | ● | ● | ● | ● | ● |
| Jitsumori^2^ | ● | ● | ● | ● | ● | ● | ● | ● |
| Imafuku^3^ | ● | ● | ● | ● | ● | ● | ● | ● |
| Cho^4^ | ● | ● | ● | ● | ● | ● | ● | ● |
| Poggi^5^ | ● | ● | ● | ● | ● | ● | ● | ● |

Risk of bias assessment was performed using the Risk Of Bias In Non-randomized Studies–of Interventions tool (ROBINS-I).^24-26^

● Low risk of bias (the study is comparable to a well-performed randomized trial with regard to this domain)

● Moderate risk of bias (the study is sound for a non-randomized study with regard to this domain but cannot be considered comparable to a well-performed randomized trial)

● Serious risk of bias (the study has some important problems in this domain)

● Critical risk of bias (the study is too problematic in this domain to provide any useful evidence on the effects of intervention.

● No information on how to base a judgment on the risk of bias for this domain.

**References**

1. Eggel B, Bernasconi M, Quibel T, Horsch A, Vial Y, Denys A, Baud D. Gynecological, reproductive and sexual outcomes after uterine artery embolization for post-partum haemorrage. Scientific reports 2021;11:833.

2. Jitsumori M, Matsuzaki S, Endo M, Hara T, Tomimatsu T, Matsuzaki S, Miyake T, Takiuchi T, Kakigano A, Mimura K, Kobayashi E, Ueda Y, Kimura T. Obstetric Outcomes of Pregnancy After Uterine Artery Embolization. International journal of women's health 2020;12:151-58.

3. Imafuku H, Yamada H, Morizane M, Tanimura K. Recurrence of post-partum hemorrhage in women with a history of uterine artery embolization. J Obstet Gynaecol Res 2020;46:119-23.

4. Cho GJ, Shim JY, Ouh YT, Kim LY, Lee TS, Ahn KH, Hong SC, Oh MJ, Kim HJ, Lee PR. Previous uterine artery embolization increases the rate of repeat embolization in a subsequent pregnancy. PLoS One 2017;12:e0185467.

5. Poggi SH, Yaeger A, Wahdan Y, Ghidini A. Outcome of pregnancies after pelvic artery embolization for postpartum hemorrhage: retrospective cohort study. Am J Obstet Gynecol 2015;213:576.e1-5.

6. Gronvall M, Tikkanen M, Paavonen J, Loukovaara M, Stefanovic V. Is there an association between postpartum hemorrhage, interventional radiology procedures, and psychological sequelae? The journal of maternal-fetal & neonatal medicine : the official journal of the European Association of Perinatal Medicine, the Federation of Asia and Oceania Perinatal Societies, the International Society of Perinatal Obstet 2021;34:1792-96.

7. Ono Y, Kariya S, Nakatani M, Ueno Y, Yoshida A, Maruyama T, Komemushi A, Tanigawa N. Clinical results of transarterial embolization for post-partum hemorrhage in 62 patients. J Obstet Gynaecol Res 2020.

8. Toguchi M, Iraha Y, Ito J, Makino W, Azama K, Heianna J, Ganaha F, Aoki Y, Murayama S. Uterine artery embolization for postpartum and postabortion hemorrhage: a retrospective analysis of complications, subsequent fertility and pregnancy outcomes. Japanese journal of radiology 2020;38:240-47.

9. Cheng HH, Tsang LL, Hsu TY, Kung CT, Ou CY, Chang CD, Tsai CC, Cheng YF, Kung FT. Transcatheter arterial embolization as first-line rescue in intractable primary postpartum hemorrhage: Assessment, outcome, and subsequent fertility. Journal of the Formosan Medical Association = Taiwan yi zhi 2017;116:380-87.

10. Inoue S, Masuyama H, Hiramatsu Y. Efficacy of transarterial embolisation in the management of post-partum haemorrhage and its impact on subsequent pregnancies. The Australian & New Zealand journal of obstetrics & gynaecology 2014;54:541-5.

11. Takeda A, Koike W, Imoto S, Nakamura H. Three-dimensional computerized tomographic angiography for diagnosis and management of intractable postpartum hemorrhage. European journal of obstetrics, gynecology, and reproductive biology 2014;176:104-11.

12. Lee HJ, Jeon GS, Kim MD, Kim SH, Lee JT, Choi MJ. Usefulness of pelvic artery embolization in cesarean section compared with vaginal delivery in 176 patients. Journal of vascular and interventional radiology : JVIR 2013;24:103-9.

13. Hardeman S, Decroisette E, Marin B, Vincelot A, Aubard Y, Pouquet M, Maubon A. Fertility after embolization of the uterine arteries to treat obstetrical hemorrhage: a review of 53 cases. Fertil Steril 2010;94:2574-9.

14. Sentilhes L, Gromez A, Clavier E, Resch B, Verspyck E, Marpeau L. Fertility and pregnancy following pelvic arterial embolisation for postpartum haemorrhage. Bjog 2010;117:84-93.

15. Fiori O, Deux JF, Kambale JC, Uzan S, Bougdhene F, Berkane N. Impact of pelvic arterial embolization for intractable postpartum hemorrhage on fertility. Am J Obstet Gynecol 2009;200:384.e1-4.

16. Gaia G, Chabrot P, Cassagnes L, Calcagno A, Gallot D, Botchorishvili R, Canis M, Mage G, Boyer L. Menses recovery and fertility after artery embolization for PPH: a single-center retrospective observational study. Eur Radiol 2009;19:481-7.

17. Chauleur C, Fanget C, Tourne G, Levy R, Larchez C, Seffert P. Serious primary post-partum hemorrhage, arterial embolization and future fertility: a retrospective study of 46 cases. Human reproduction (Oxford, England) 2008;23:1553-9.

18. Eriksson LG, Mulic-Lutvica A, Jangland L, Nyman R. Massive postpartum hemorrhage treated with transcatheter arterial embolization: technical aspects and long-term effects on fertility and menstrual cycle. Acta radiologica (Stockholm, Sweden : 1987) 2007;48:635-42.

19. Shim JY, Yoon HK, Won HS, Kim SK, Lee PR, Kim A. Angiographic embolization for obstetrical hemorrhage: effectiveness and follow-up outcome of fertility. Acta Obstet Gynecol Scand 2006;85:815-20.

20. Descargues G, Mauger Tinlot F, Douvrin F, Clavier E, Lemoine JP, Marpeau L. Menses, fertility and pregnancy after arterial embolization for the control of postpartum haemorrhage. Human reproduction (Oxford, England) 2004;19:339-43.

21. Salomon LJ, deTayrac R, Castaigne-Meary V, Audibert F, Musset D, Ciorascu R, Frydman R, Fernandez H. Fertility and pregnancy outcome following pelvic arterial embolization for severe post-partum haemorrhage. A cohort study. Human reproduction (Oxford, England) 2003;18:849-52.

22. Ornan D, White R, Pollak J, Tal M. Pelvic embolization for intractable postpartum hemorrhage: long-term follow-up and implications for fertility. Obstet Gynecol 2003;102:904-10.

23. Picone O, Salomon LJ, Ville Y, Kadoch J, Frydman R, Fernandez H. Fetal growth and Doppler assessment in patients with a history of bilateral internal iliac artery embolization. The journal of maternal-fetal & neonatal medicine : the official journal of the European Association of Perinatal Medicine, the Federation of Asia and Oceania Perinatal Societies, the International Society of Perinatal Obstet 2003;13:305-8.

24. Sterne JA, Hernan MA, Reeves BC, Savovic J, Berkman ND, Viswanathan M, Henry D, Altman DG, Ansari MT, Boutron I, Carpenter JR, Chan AW, Churchill R, Deeks JJ, Hrobjartsson A, Kirkham J, Juni P, Loke YK, Pigott TD, Ramsay CR, Regidor D, Rothstein HR, Sandhu L, Santaguida PL, Schunemann HJ, Shea B, Shrier I, Tugwell P, Turner L, Valentine JC, Waddington H, Waters E, Wells GA, Whiting PF, Higgins JP. ROBINS-I: a tool for assessing risk of bias in non-randomised studies of interventions. BMJ 2016;355:i4919.

25. Danna SM, Graham E, Burns RJ, Deschenes SS, Schmitz N. Association between Depressive Symptoms and Cognitive Function in Persons with Diabetes Mellitus: A Systematic Review. PLoS One 2016;11:e0160809.

26. ROBINS-I detailed guidance (2016). <https://www.riskofbias.info/welcome/home/current-version-of-robins-i/robins-i-detailed-guidance-2016>. (accessed 09/20/2020).
